# From Sea to Therapy: Marine Biomaterials for Drug Delivery and Wound Healing

**DOI:** 10.3390/ph18081093

**Published:** 2025-07-23

**Authors:** Mansi Chilwant, Valentina Paganini, Mariacristina Di Gangi, Sofia Gisella Brignone, Patrizia Chetoni, Susi Burgalassi, Daniela Monti, Silvia Tampucci

**Affiliations:** 1 Department of Pharmacy, University of Pisa, Via Bonanno 33, 56126 Pisa, Italy; m.chilwant@studenti.unipi.it (M.C.); valentina.paganini@phd.unipi.it (V.P.); mariacristina.digangi@phd.unipi.it (M.D.G.); sofiagisella.brignone@farm.unipi.it (S.G.B.); patrizia.chetoni@unipi.it (P.C.); susi.burgalassi@unipi.it (S.B.); daniela.monti@unipi.it (D.M.); 2 Interdepartmental Center of Marine Pharmacology, University of Pisa, Via Bonanno 6, 56126 Pisa, Italy

**Keywords:** marine biomaterials, polysaccharides, proteins, peptides, sustainable drug delivery, wound healing, biomass, bioeconomy, blue biotechnology, industrial by-products, chitosan, alginate, collagen

## Abstract

Marine biomass represents a valuable yet underexploited resource for the development of high-value biomaterials. Recent advances have highlighted the significant potential of marine-derived polysaccharides, proteins, and peptides in biomedical applications, most notably in drug delivery and wound healing. This review provides a comprehensive synthesis of current research on the extraction, processing and pharmaceutical valorization of these biopolymers, with a focus on their structural and functional properties that allow these materials to be engineered into nanocarriers, hydrogels, scaffolds, and smart composites. Key fabrication strategies such as ionic gelation, desolvation, and 3D bioprinting are critically examined for their role in drug encapsulation, release modulation, and scaffold design for regenerative therapies. The review also covers preclinical validation, scale-up challenges, and relevant regulatory frameworks, offering a practical roadmap from sustainable sourcing to clinical application. Special attention is given to emerging technologies, including stimuli-responsive biomaterials and biosensor-integrated wound dressings, as well as to the ethical and environmental implications of marine biopolymer sourcing. By integrating materials science, pharmaceutical technology and regulatory insight, this review aims to provide a multidisciplinary perspective for researchers and industrial stakeholders seeking sustainable and multifunctional pharmaceutical platforms for precision medicine and regenerative therapeutics.

## 1. Introduction

In the current landscape of pharmaceutical development, characterized by an expanding pipeline of small-molecule and biologic drug candidates, the need for advanced delivery systems to overcome challenges in bioavailability, stability, and patient compliance is more pressing than ever. Among the most promising frontiers in this field is the valorization of marine biomass, particularly seafood processing by-products, as a source of bioactive polysaccharides and proteins for applications in drug delivery and wound healing [1,2,3].

Covering over 70% of the Earth’s surface, oceans represent a vast and still insufficiently exploited source of biologically active materials. According to recent estimates, global seafood processing activities generate approximately 30 million tons of by-products annually, such as fish skin and shells, which can account for up to 85% of the total marine biomass depending on the species and represent a primary focus due to their abundance and low cost (Roy et al., 2023 [4]). Marine biomass, including seafood processing by-products as well as primary resources like seaweed and fish connective tissues, offers an abundant supply of structurally diverse biopolymers with therapeutic potential [3]. In this review, the term marine biomass refers to both categories, with a specific focus on biomaterials relevant to drug delivery and wound healing. Although marine lipids and alkaloids display interesting pharmacological properties, their application in delivery systems is often limited by issues such as chemical instability, complex extraction, and low formulation versatility. In contrast, marine-derived polysaccharides (e.g., chitin, chitosan, alginate, fucoidan, and carrageenan) and protein (e.g., collagen, gelatin) exhibit excellent biocompatibility, biodegradability, and tunable physicochemical properties as well as adaptability to be included in drug delivery systems [3,5,6]. These features make them particularly suitable for biomedical use and justify the central role in the present review.

Despite their high biological potential and environmental advantages, marine biomaterials remain underexploited in the pharmaceutical field due to several factors, including raw material variability, limited standardization, and lack of regulatory harmonization. Although preclinical data are promising, many compounds are still under investigation, with only a few having reached advanced stages of translational development. To better assess their pharmaceutical relevance, it is crucial to compare marine-derived biomaterials with synthetic and non-marine alternatives. Synthetic polymers such as PEG, PVA, and polycaprolactone (PCL) are widely used in biomedical applications thanks to their reproducible mechanical properties, batch-to-batch consistency, and tunable physicochemical and degradation profiles. These materials support advanced processing methods, including electrospinning or 3D printing, allowing for precise modulation of the scaffold architecture and sustained drug release. However, they often lack intrinsic biological activity and may raise concerns related to immunogenicity, limited biodegradability, or the use of toxic crosslinkers [7,8,9].

In contrast, marine biomaterials combine biocompatibility and biodegradability with multifunctional bioactivity that can enhance therapeutic efficacy without additional chemical agents. Besides the above-mentioned sustainability advantages, issues like compositional variability, limited scalability, and challenges in standardization and regulatory approval still limit their broader application.

**Figure 1:**
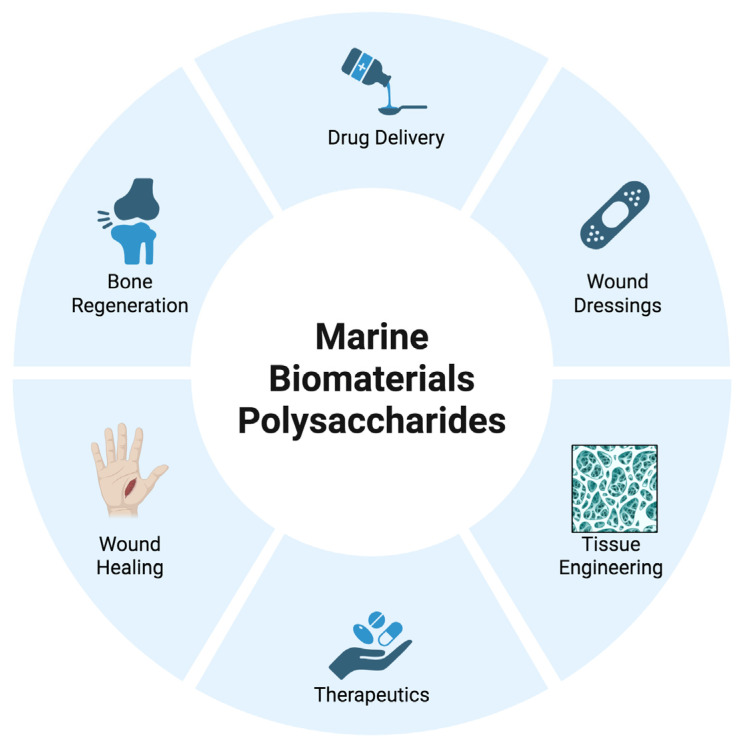
Schematic representation of the main biomedical applications of marine biomaterials. These include drug delivery, wound dressing, tissue engineering, therapeutics, wound healing, and bone regeneration (Created in BioRender. Chilwant, M. (2025) https://BioRender.com/zq9m8ff).

In this context, marine biopolymers are currently being investigated in a broad range of biomedical applications, including drug delivery, wound dressings, tissue engineering, therapeutics, wound healing, and regenerative medicine, as summarized in Figure 1. Moreover, their properties enable the development of nanostructured drug delivery systems capable of targeted, controlled, and sustained release of therapeutic agents, such as growth factors, antimicrobial molecules, and anti-inflammatory compounds. Therefore, they represent a valuable pharmaceutical source that can enhance therapeutic efficacy while reducing systemic side effects [6].

In addition to their functional versatility, these biomaterials offer significant environmental advantages. Being naturally abundant and renewable, they align with the growing emphasis on sustainability in pharmaceutical innovation. Their compatibility with mild and scalable fabrication techniques, such as ionic gelation, emulsion–solvent evaporation, and desolvation, allows for precise control over particle size, drug loading, and release kinetics. This adaptability supports their customization across a broad range of clinic indications, from acute wounds to chronic inflammatory diseases [3].

Each marine-derived biopolymer provides unique therapeutic advantages. Chitosan, derived from crustacean exoskeletons, exhibits intrinsic antibacterial and wound-healing properties. Alginate, extracted from brown algae, forms hydrogels with excellent moisture-retaining capacity. Marine collagen, isolated from fish connective tissues, is highly biocompatible and has low immunogenicity, making it ideal for tissue regeneration [10,11,12].

Preclinical studies have demonstrated the efficacy of these systems in managing chronic wounds and infections. Chitosan-based nanoparticles stimulate fibroblast proliferation and collagen synthesis, both essential for tissue repair. Alginate nanoparticles, particularly when loaded with bioactive agents such as curcumin, possess antimicrobial and anti-inflammatory properties. Similarly, nanoparticles based on marine collagen and gelatin facilitate cell migration and extracellular matrix (ECM) formation, processes critical for functional tissue regeneration [13,14,15].

**Figure 2:**
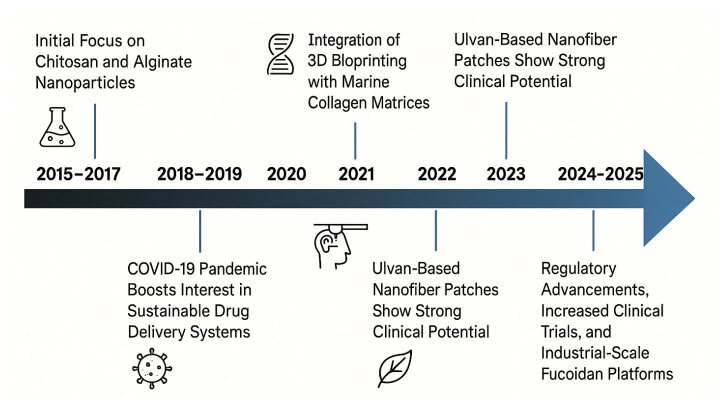
Timeline of key advances in marine biomaterials for wound healing and drug delivery in the period of 2015–2025 (Created in BioRender. Chilwant, M. (2025) https://BioRender.com/9yeslx4).

Over the past decade, the biomedical exploitation of these materials has progressed through well-defined phases, as described in the diagram of Figure 2. From 2015 to 2017, initial investigations focused on harnessing the intrinsic properties of chitosan and alginate for drug delivery and wound management [15,16,17,18]. Between 2018 and 2019, attention turned to marine collagen-based scaffolds, which offered clear advantages over mammalian analogues in terms of lower immunogenicity and zoonotic risk. These scaffolds, typically derived from fish skin or connective tissue, have shown the ability to mimic the extracellular matrix, facilitating cell adhesion, migration, and tissue regeneration, especially relevant in oral mucosa and dermal repair contexts [19,20]. In response to the COVID-19 pandemic in 2020, the pharmaceutical industry intensified its focus on sustainable drug delivery solutions. Marine-derived polymers gained attraction for their ability to enhance drug solubility and targeting, while supporting global imperatives for renewable materials [21]. The research further improved with the innovation of smart biomaterials. By 2021, marine-derived hydrogels integrated with nanoparticles and biosensors enabled the development of multifunctional wound dressings capable of real-time monitoring of wound conditions (e.g., pH, temperature, exudate) while simultaneously accelerating healing [22,23]. Recent advances in nanotechnology have further expanded the therapeutic potential of these materials. Marine-based nanoparticles can be engineered to incorporate additional functional elements, such as silver nanoparticles or hyaluronic acid, to enhance antimicrobial efficacy, stimulate tissue regeneration, and improve therapeutic outcomes [12,24,25].

Building on these advancements, 2022 marked the integration of marine collagen matrices with 3D bioprinting technologies to fabricate highly structured scaffolds, which closely replicate native tissue architecture and are biologically compatible, for application in skin, cartilage, and bone regeneration [19,26].

In 2023, researched focused on ulvan, a sulfated polysaccharide derived from green algae (*Ulva* spp.), which was formulated into electrospun nanofiber mats, that, especially when combined with marine gelatin, demonstrated enhanced wound contraction and epithelial regeneration in preclinical models [27,28].

Most recently (2024–2025), the field has entered a phase of clinical maturation and regulatory progress. Several marine biopolymers, notably fucoidan-based formulations, are undergoing scale-up for industrial manufacturing and early-phase clinical trials for diverse therapeutic indications. These advances underscore the translational potential of marine-derived biomaterials, not only as excipients or carriers but also as bioactive agents with intrinsic therapeutic properties [29,30].

Taken together, the integration of marine-derived biomaterials into drug delivery systems represents a promising strategy for the development of next-generation pharmaceutical technologies. Differentiating itself from the existing literature by providing a structured and chronological analysis of the scientific and technological progress in the field in the last ten years, this review offers an integrated and up-to-date overview of marine-derived biomaterials with a particular focus on their translational potential, scalability, and regulatory readiness. Advances in fabrication technologies (e.g., 3D bioprinting, nanostructured systems), the emergence of novel materials such as ulvan, and increasing clinical and industrial interest are examined in light of their implications for drug delivery and wound healing. By addressing both carrier and bioactive roles of these biomaterials, the review contributes to a better understanding of their role in the development of more sustainable, personalized, and effective therapeutic strategies.

## 2. Polysaccharides and Protein-Based Biomaterials from Marine Source

The following sections provide an overview of the main marine-derived biopolymers, focusing on their structural features, physicochemical properties, and biomedical applications. Special attention is given to their role as enabling materials in the design of advanced drug delivery systems and regenerative platforms. A schematic summary of the key fabrication strategies associated with these biopolymers is provided in Table 1, to offer a comparative overview of their technological processing, while preclinical and clinical studies reported in the following sub-paragraphs are summarized in Table 2.

### 2.1. Chitosan

Chitosan, a cationic polysaccharide obtained from the deacetylation of chitin, derived from crustacean shells, is among the most promising marine-derived biomaterials for targeted drug and gene delivery [31]. Its intrinsic properties, including mucoadhesiveness, biocompatibility, biodegradability, and the ability to be chemically modified, make it an ideal platform for the development of nanostructured drug delivery systems. These features provide a strong and versatile platform for application in nanomedicine [10].

**Figure 3:**
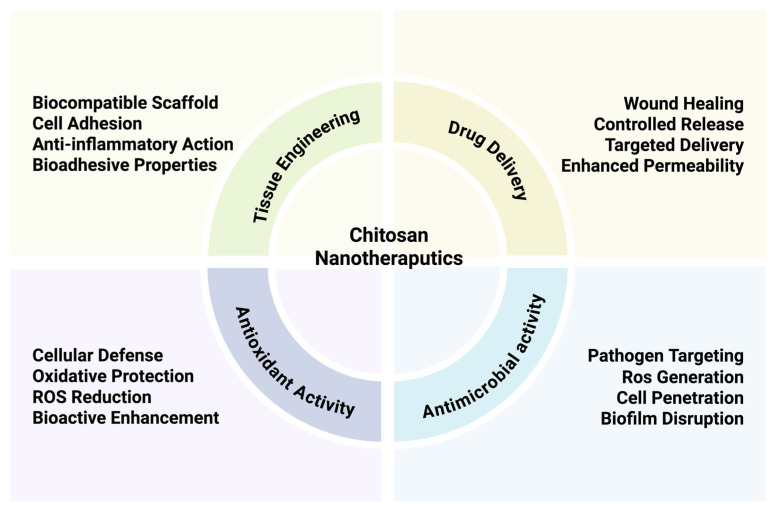
Therapeutic applications of chitosan nanotherapeutics. The figure highlights four key areas (tissue engineering, drug delivery, antioxidant activity, and antimicrobial activity), each associated with specific properties (Created in BioRender. Chilwant, M. (2025) https://BioRender.com/c6hourh).

Chitosan-based nanotherapeutics offer multiple advantages across a broad spectrum of therapeutic applications, as illustrated in Figure 3. One of their most prominent uses lies in drug delivery, where they enable controlled and targeted drug release, protect encapsulated agents from degradation, and improve permeation across biological barriers. These features are especially important in case of the delivery of heat- and pH-sensitive therapeutic agents like peptides, proteins, and growth factors. Chitosan nanoparticles are commonly prepared via ionic gelation, which involves crosslinking chitosan with polyanionic agents such as tripolyphosphate (TPP). This method is simple, conducted under mild conditions, and preserves the structural integrity of delicate therapeutic agents [32]. It is often preferred over other techniques, such as solvent evaporation, due to its superior compatibility with labile biomolecules and generally higher encapsulation efficiency. Additionally, chitosan nanoparticles can be engineered to respond to specific stimuli, such as pH changes or enzymatic activity, enabling site-specific release at the target site. Their positive charge facilitates interaction with negatively charged cell membranes and mucosal surfaces, thereby improving mucoadhesion. This property prolongs the residence time of the formulation at the site of absorption, enhancing drug bioavailability and therapeutic efficacy in mucosal drug delivery systems.

Beyond their delivery functions, chitosan nanocarriers exhibit intrinsic antimicrobial and antioxidant properties that further expand their therapeutic potential. As highlighted in Figure 3, chitosan nanoparticles can target pathogens by disrupting microbial membranes penetrating biofilms, and potentially inducing localized ROS generation, thereby inhibiting microbial proliferation. These properties are particularly valuable for localized therapies targeting infected or inflamed tissues.

At the same time, chitosan contributes to cellular defense by scavenging reactive oxygen species (ROS), reducing oxidative stress, and enhancing the bioactivity of co-delivered therapeutic agents [24]. This dual antimicrobial/antioxidant effect is especially relevant for applications in chronic wounds, inflammatory disorders, or oxidative-stress-related pathologies. Notably, chitosan is effective even against multidrug-resistant strains and contributes to pain reduction and patient comfort due to its gentle adhesion and ease of removal [33]. The versatility of chitosan platforms is further demonstrated by their integration with other bioactive components, such as silver nanoparticles or chlorhexidine, and their functionalization with molecules like hyaluronic acid to enhance tissue affinity and regenerative performance [11]. These modifications are particularly relevant for applications in tissue engineering and wound healing (Figure 3), where bioadhesion, cell proliferation, and anti-inflammatory effects play key roles. Together, these multifunctional properties position chitosan nanoparticles as a highly adaptable platform not only for systemic or mucosal drug delivery but also for localized therapies requiring antimicrobial action, oxidative protection, and tissue regeneration.

Chitosan also plays a pivotal role in wound healing thanks to its combined hemostatic, antimicrobial, immunomodulatory, and regenerative properties. Several studies confirm the regenerative potential of chitosan and marine-derived biomaterials. A preclinical study demonstrated that chitosan-based hydrogels enriched with aloe vera significantly accelerated wound healing in a full-thickness rat model, achieving complete wound closure within 14 days, along with enhanced epidermal thickness and reduced inflammation compared to controls and standard treatments such as silver sulfadiazine [23]. When applied to skin lesions, chitosan-based nanoparticles interact with components of the extracellular matrix (ECM), binding to negatively charged glycosaminoglycans; this interaction promotes cell adhesion, stimulates fibroblast proliferation, and facilitates cell migration, essential for tissue regeneration. The cationic nature of chitosan enhances angiogenesis, the formation of new blood vessels, by improving cell–matrix interactions and supporting the delivery of oxygen and nutrient to the wound site. Moreover, chitosan’s immunomodulatory activity contributes to the regulation of the inflammatory response by suppressing pro-inflammatory cytokines, thereby preventing chronic inflammation and facilitating tissue repair. In addition, chitosan can release bioactive oligosaccharides that act as signaling molecules, enhancing the cellular responses involved in tissue regeneration and activating keratinocytes and fibroblasts, the main cell types involved in epidermal and dermal repair, respectively [11,24,25]. Chitosan also stimulates immune cells such as macrophages, promoting the release of cytokines and macrophage inflammatory protein-2 (MIP-2), which in turn enhances epithelial proliferation and collagen synthesis [10,24]. In parallel, chitosan has been shown to modulate the expression of transforming growth factor β (TGF-β), a key regulator of extracellular matrix remodeling and fibroblast activation, further supporting re-epithelialization and improving the tensile strength and structural integrity of the regenerated tissue [26]. Chitosan has also been shown to stimulate fibroblast proliferation and collagen synthesis in a dose-dependent manner. This effect is further supported by the upregulation of key growth factors, such as TGF-β, which promote extracellular matrix (ECM) production and tissue remodeling. Interestingly, chitosan with a high degree of deacetylation has been shown to promote greater fibroblast activity, emphasizing the critical role of material selection to achieve optimal biological effects [11]. Functionalization with hyaluronic acid further amplifies chitosan’s therapeutic effects, enhancing cell adhesion, re-epithelialization, and hydration of the wound bed [22]. Such chitosan-based dressings prevent scar formation and support the regeneration of a structurally and functionally competent skin barrier. Its hygroscopic nature helps maintain a moist wound environment, conducive to cell proliferation and ECM deposition. This makes chitosan particularly effective in treating both acute and chronic wounds, including those complicated by infection or poor vascularization.

As stated above, chitosan also exhibits hemostatic activity that directly contributes to its effectiveness in wound-healing applications. This pro-coagulant capacity facilitates rapid bleeding control at the injury site, creating a favorable environment for subsequent tissue repair. In a clinical study involving patients with coagulation disorders or on anticoagulant therapy undergoing tooth extractions, chitosan-based dressings significantly reduced the mean hemostasis time to 9.80 ± 15.49 min, compared to 44.23 ± 22.41 min with conventional gauze, confirming their superior hemostatic efficacy [34]. A chitosan sponge further decreased the hemostatic time to 38 ± 8.7 s, inducing faster clotting compared to control materials [35]. Additionally, chitosan-based hydrogels demonstrated up to a 77% reduction in bleeding volume compared to controls [36] and in vitro assays confirmed that the pro-coagulant activity produced a reduction in clotting time by approximately 40% with respect to untreated blood [19]. Notably, a nanoengineered chitosan–graphene scaffold produced an 87% reduction in clotting time compared to commercial hemostatic agent QuikClot [20].

In this context, it is important to mention chitin, the natural precursor of chitosan, which also plays a significant role in hemostasis and wound healing, due to its antimicrobial, regenerative, and pro-proliferative properties [10,11]. Chitin interacts electrostatically with red blood cells, promoting clot formation, and in its hydrogel form, it mimics the extracellular matrix (ECM), providing a favorable environment for tissue integration. Chitin-based dressings help reduce local inflammation, thus promoting tissue repair [7]. When combined with other polymers, chitin/chitosan composites exhibit enhanced mechanical strength and bioactivity, making them strong candidates for next-generation wound dressings and tissue engineering applications [10,11,25].

### 2.2. Alginate

Alginate is a biopolymer mainly extracted from brown algae like *Laminaria*, *Ascophyllum*, or *Macrocystis* using alkaline extraction followed by precipitation and purification. It is an anionic polysaccharide composed of mannuronic acid (M) and guluronic acid (G) monomers linked through β-(1→4) and α-(1→4) glycosidic bonds, which form M-blocks, G-blocks, and alternating MG-block sequences. The degree of polymerization and M/G ratio varies significantly depending on the source, affecting mechanical properties and gelling behavior. Its chemical structure greatly influences its physicochemical and biological properties, such as gelation capacity, water retention, and biodegradability [37,38]. Alternatively, microbial production via Pseudomonas or Azotobacter allows for a more controlled composition and higher G-block content, resulting in stiffer, more defined gels, but at a higher production cost and with scale-up challenges [39].

**Figure 4:**
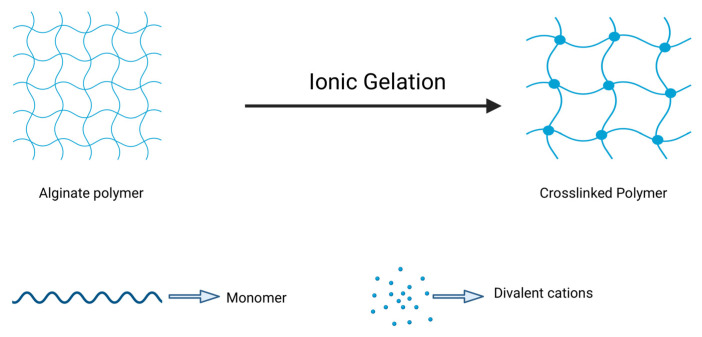
Ionic gelation techniques for alginate polymer. Schematic representation of the ionic gelation mechanism of alginate. Upon exposure to divalent cations (e.g., Ca^2+^), the linear alginate polymer chains undergo crosslinking, forming a three-dimensional hydrogel network. This transformation is driven by the interaction between the carboxyl groups on guluronic acid residues and the cations, resulting in junction zones that stabilize the structure. The degree of crosslinking, and thus the final mechanical and swelling properties, depends on factors such as cation type, concentration, and the M/G ratio of the alginate (Created in BioRender. Chilwant, M. (2025) https://BioRender.com/1vr8dgg).

One of the distinguishing features of alginate is its ability to undergo gelation upon interaction with divalent cations such as calcium (Ca^2+^) or barium (Ba^2+^), which crosslink G-blocks to produce a three-dimensional network capable of retaining water and providing structural stability. This mild, reversible, and tunable gelation process is widely exploited to produce alginate-based nanogels for drug delivery and wound-healing applications by adjusting parameters such as alginate concentration, type and concentration of crosslinkers, and environmental factors like pH and ionic strength (Figure 4) [40].

The ionotropic gelation technique enables the production of alginate nanoparticles with a high surface area and structural uniformity, suitable for encapsulation and sustained delivery of bioactive compounds such as antibiotics, anti-inflammatory agents, and growth factors. These nanosystems are biocompatible and capable of minimizing systemic side effects by enabling localized and controlled drug release in response to environmental stimuli like pH and ionic strength variations. Such features make alginate an attractive material to develop advanced nanocarriers for biomedical application [40,41]. However, while the gelation is advantageous for biomedical use, alginate lacks specific cell adhesion motifs, which limits cell–material interactions. This issue can be mitigated by conjugating alginate with peptides such as RGD (Arg-Gly-Asp) or blending it with gelatin, enhancing cell anchorage and viability [39].

In addition to its role in drug delivery, alginate is widely used in wound healing, particularly as hydrogels and dressings for acute and chronic lesions. The hydrogel form of alginate can be synthesized using various crosslinking methods and its structure resembles that of extracellular matrices of living tissues. Upon contact with wound exudate, it forms a hydrophilic gel that maintains a moist environment, supports cellular migration, and promotes granulation tissue formation. This gel layer not only facilitates epithelial regeneration but also reduces the risk of scarring. When loaded with bioactive agents, alginate hydrogels combine moisture control with therapeutic functions such as antimicrobial activity and inflammation modulation, enhancing localized treatment while minimizing systemic exposure [37,38].

Alginate’s exceptional fluid absorption capacity, making it capable of absorbing 20 to 30 times its own weight in wound exudate, contributes significantly to its performance as a wound dressing. Clinical evidence underscores its regenerative potential: in burn wounds, the healing time was reduced to 7 ± 3.5 days, compared to 14 ± 4.2 days with conventional treatments [42]. In a prospective randomized controlled trial involving full-thickness pressure ulcers, 74% of patients treated with alginate experienced at least a 40% wound area reduction within four weeks, compared to 42% in the control group, where healing durations often exceeded eight weeks. Further studies with powered-release alginate fiber dressings reported a mean healing time of 10.6 days. A recent meta-analysis of randomized controlled trials confirmed these outcomes, showing consistently faster wound healing and improved clinical results compared to standard approaches [43].

Moreover, alginate’s hemostatic properties, derived from calcium-mediated interactions with blood components, make it particularly effective in post-surgical and trauma settings. Its ability to be formulated into dressings with a customizable thickness, gelation rate, and drug-loading capacity allows for personalized wound management. Recent evidence shows that bioactive-loaded alginate formulations enhance healing by reducing the infection risk, accelerating tissue regeneration, and improving patient recovery [44].

### 2.3. Marine Collagen and Gelatin

**Figure 5:**
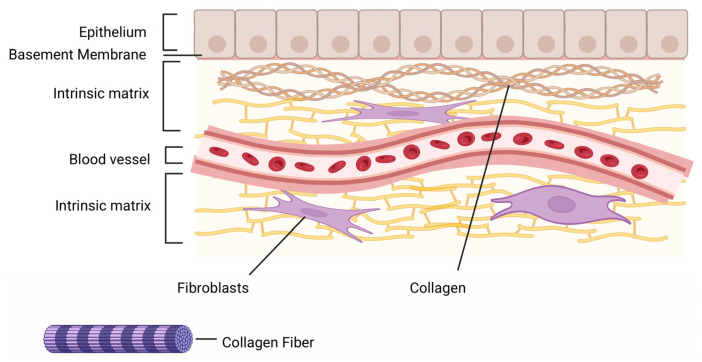
Key biofunctional properties of marine collagen peptides. Marine-derived collagen peptides exhibit a broad range of biological activities, including high biocompatibility, minimal immunogenicity, and enrichment in type I collagen. Their multifunctional profile supports antimicrobial, antioxidant, and anti-aging effects, along with improved hydration and tissue integration, making them ideal candidates for cosmetic, pharmaceutical, and biomedical applications (Created in BioRender. Chilwant, M. (2025) https://BioRender.com/9f9hxaz).

**Figure 6:**
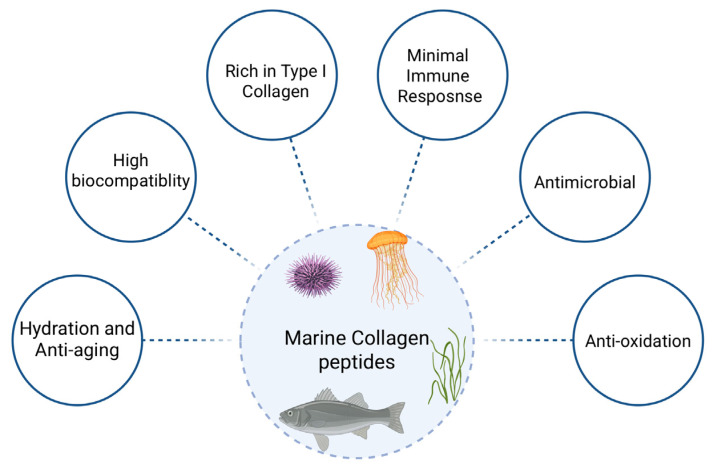
Schematic representation of the skin extracellular matrix (ECM) microenvironment. The diagram illustrates the hierarchical organization of the skin, including the epithelium, basement membrane, and intrinsic matrix layers. Fibroblasts embedded within the collagen-rich ECM contribute to tissue homeostasis, repair, and remodeling. Collagen fibers provide structural integrity and serve as scaffolds for cellular adhesion, proliferation, and migration. The presence of blood vessels highlights the vascular support essential for nutrient delivery and waste removal (Created in BioRender. Chilwant, M. (2025) https://BioRender.com/h5pjv1k).

Marine collagen, an insoluble fibrous structural protein derived from the connective tissues of marine organisms, particularly fish skin and scales, and its denatured form, gelatin, are gaining increasing attention for biomedical applications. Their high biocompatibility, low immunogenicity, and biodegradability render them highly suitable for use in tissue engineering, wound healing, and drug delivery systems (Figure 5) [45,46,47]. As a major component of the ECM, collagen plays a pivotal role in cellular signaling, tissue organization, and structural repair (Figure 6). Upon denaturation, collagen converts into gelatin, which retains most of the bioactive properties while exhibiting greater solubility and ease of processing, primarily due to its inherent gelling ability.

In drug delivery, marine collagen and gelatin are employed to formulate nanoparticles through methods such as desolvation, coacervation, emulsification, or spray drying. These approaches enable the encapsulation of therapeutic agents, including growth factors, antimicrobials, and small-molecule drugs, within the polymer matrix, allowing for controlled release, targeted delivery, and prolonged bioactivity. For instance, desolvation involves the addition of a non-solvent to precipitate collagen nanoparticles, while coacervation exploits electrostatic interactions or pH changes to induce phase separation. These approaches yield nanoparticles with a precisely defined size, morphology, and encapsulation efficiency, thus confirming their role as effective nanocarriers in drug delivery applications [48].

To enhance mechanical stability and modulate the release profile, crosslinking agents such as glutaraldehyde or genipin are commonly employed, particularly in gelatin-based systems. These nanocarriers exhibit an excellent encapsulation efficiency and tunable degradation rates, making them suitable for injectable formulations or implantable delivery platforms in regenerative medicine.

Concurrently, marine collagen and gelatin play a prominent role in wound healing and tissue regeneration. Marine collagen peptides, rich in type I collagen, promote fibroblast stimulation, angiogenesis, antioxidant protection, and moisture retention, all of which are crucial for restoring skin integrity and accelerating dermal recovery [47].

Ideal for wound healing, tissue regeneration, and injectable formulations, their great biocompatibility and low immunoreactivity guarantee safe integration into human tissues. Known for their hydration-boosting and anti-ageing benefits, marine collagen peptides improve overall dermal health, increase skin elasticity, and reduce wrinkle formation by encouraging moisture retention and stimulating fibroblast activity. Additionally, their antibacterial properties and capacity to maintain hydration make them ideal for treating chronic or infected wounds [45,47].

These multifunctional properties support the use of marine collagen peptides as bioactive ingredients in nutraceuticals, cosmetics, and pharmaceutical products. Compared to mammalian collagen, marine collagen offers superior solubility under physiological conditions and a lower risk of zoonotic disease transmission, supporting its role in clinical and preclinical applications [45].

Recent clinical data further confirm its potential. In a triple-blind randomized controlled trial involving 50 women, daily oral supplementation with 10 g of marine-derived collagen over 12 weeks resulted in a 35% reduction in facial wrinkles, 14% increase in skin hydration, 25% improvement in firmness, and 22% boost in skin radiance compared to the placebo [46]. Although focused on cosmetic endpoints, these data reflect a structurally enhanced ECM integrity, collagen synthesis, and dermal repair, mechanisms equally relevant to wound healing.

Collagen-based scaffolds, designed to mimic the native ECM, offer a supportive three-dimensional architecture as well as an optimal microenvironment for cellular function. Their porous structure and spatial organization favor oxygen diffusion, nutrient transport, and cellular infiltration, ensuring optimal integration of the engineered tissue into the host tissue. Functionalization of these scaffolds with bioactive molecules, including peptides and growth factors, further enhances their regenerative capabilities, facilitating not only cutaneous wound healing but also the regeneration of complex tissues like bone and cartilage.

Recent advances have explored the use of 3D bioprinting to fabricate collagen-based structures with tailored mechanical and biological properties. These technologies enable the development of personalized scaffolds for organ repair, orthopedic reconstruction, and skin regeneration, greatly expanding the therapeutic application of marine collagen in regenerative medicine [49,50].

Similarly, marine collagen-based scaffolds derived from sea urchins showed efficacy in wound healing in vivo. In a sheep wound-healing model, treatment with collagen-based skin scaffolds (CBSSs) led to enhanced keratinocyte migration, increased granulation tissue, and a reduced inflammatory response by day 14. Moreover, there was a marked upregulation of VEGF-A, a key mediator of angiogenesis, and hKER, a marker of hair follicle regeneration, highlighting the role of marine collagen in orchestrating essential cellular processes for effective skin repair [51].

### 2.4. Fucoidan

Fucoidan, a sulfated polysaccharide primarily derived from brown marine algae, has emerged as a promising biomaterial for a wide range of biomedical applications, thanks to its unique structural complexity and multifunctional bioactivity. Composed mainly of L-fucose and sulfate ester groups, fucoidan exhibits biological properties that vary significantly depending on factors such as molecular weight, sulfation pattern, monosaccharide composition, and extraction conditions [52]. These structural nuances influence its pharmacological effects, which include anticoagulant, antithrombotic, antiangiogenic, anti-inflammatory, antiviral, and antioxidant activities [53].

In addition, fucoidan has demonstrated prebiotic properties by modulating the growth and metabolic activity of beneficial gut microbiota such as *Lactobacillus rhamnosus*, enhancing their antibacterial potential and supporting intestinal health [54]. This opens up further avenues for its application in gut-targeted therapies and functional food formulations.

Beyond these traditional roles, fucoidan is increasingly being explored as a functional agent in drug delivery systems. Indeed, fucoidan can be engineered into various nanostructures—such as nanoparticles, microspheres, nanofibers, and hydrogels—tailored to improve its solubility, chemical stability, and bioavailability. These delivery platforms allow the controlled release of therapeutic agents, including anticancer drugs, anti-inflammatory compounds, or peptides. Its ability to bind to specific receptors, such as P-selectin and CD44, commonly overexpressed in tumor and inflamed tissues, confers it with intrinsic targeting capabilities. Recent studies have emphasized the utility of fucoidan as a ligand for receptor-mediated uptake and as a modulator of immune receptor pathways (e.g., TLRs, SRs, lectins), thereby enhancing the efficacy of nanocarrier-based therapeutics, especially in cancer and immunotherapy [55].

In this context, a variety of formulation strategies have been proposed to exploit fucoidan’s therapeutic potential. For example, Lai et al. [56] developed fucoidan-PLGA nanoparticles (FPNs) loaded with docetaxel (DTX), demonstrating enhanced colloidal stability, tunable drug release, and superior anticancer efficacy against triple-negative breast cancer cells compared to PLGA-only formulations. The FPNs showed a higher encapsulation efficiency, reduced burst release, and long-term physical stability (up to 4 weeks at 4 °C), with the fucoidan shell imparting both a negative surface charge and bioactivity.

Furthermore, computational approaches have recently been introduced to optimize fucoidan–receptor binding profiles, facilitating the rational design of drug delivery systems with disease-specific targeting. Docking simulations and AI-based prediction models suggest that selectins, lectins, and CD44 are preferential fucoidan targets, providing a mechanistic basis for its selective bioactivity in cancer and inflammatory diseases [55].

Simultaneously, fucoidan has shown strong potential in wound-healing applications, where its anti-inflammatory, antioxidant, and angiogenesis-regulating functions support tissue repair and regeneration. Incorporated into hydrogels or scaffolds, it helps maintain a moist wound environment, modulates immune responses, and protects against oxidative stress, thereby accelerating healing.

In experimental models utilizing murine full-thickness skin wounds, the topical administration of 1.2% fucoidan markedly accelerated wound healing, exhibiting a significant disparity in the rate of wound closure from day 6 post-injury with respect to control groups. Histological evaluations demonstrated a pronounced increase in granulation tissue thickness (~30%) on days 7 and 14 in the fucoidan-treated cohort. Furthermore, collagen deposition was observed to be significantly elevated by approximately 40% in treated wounds, corroborating the promotion of extracellular matrix formation [57].

Wen et al. [57] provided compelling evidence that fucoidan enhances angiogenesis and promotes vascular maturation by activating the AKT/Nrf2/HIF-1α signaling pathway. In vivo, fucoidan treatment accelerated neovessel formation and increased the density and organization of CD31^+^/α-SMA^+^ vascular structures, particularly in the central wound region. In vitro, fucoidan stimulated the proliferation of HUVECs and significantly restored their ability to form tubular networks under oxidative stress conditions, accompanied by upregulation of angiogenesis-related proteins including eNOS, VEGF, Nrf2, and HIF-1α.

Innovative composite materials incorporating fucoidan into hydrogels or scaffolds have also been explored. A recent study evaluated a gelatin/oxidized carboxymethyl cellulose hydrogel loaded with fucoidan derived from *Ecklonia cava*, which exhibited robust antioxidant activity, promoted macrophage polarization, and stimulated fibroblast-mediated collagen production. The resulting hydrogel demonstrated excellent cytocompatibility, mechanical stability, and prolonged release behavior, highlighting its suitability as an advanced wound dressing [58].

The ability of fucoidan to form bioactive composite materials, and its compatibility with other polymers such as chitosan, alginate, gelatin, or PLGA, further expands its application potential in designing multifunctional systems for controlled delivery, tissue engineering, and regenerative medicine.

These composite systems exploit synergistic effects, fucoidan’s bioactivity, and the carrier’s mechanical or structural properties, enhancing therapeutic outcomes.

### 2.5. Carrageenan

Carrageenan, particularly in its kappa form, is a marine-derived sulfated polysaccharide that combines biocompatibility, biodegradability, and chemical versatility, making it highly suitable for both wound healing and drug delivery applications. Its gelling behavior, mucoadhesiveness, and ionic responsiveness allow the formation of diverse systems, including hydrogels, films, microspheres, and nanocomposites tailored for biomedical purposes [59].

In wound management, carrageenan-based systems, such as gels, hydrogels, and porous composite dressings, have demonstrated efficacy in promoting tissue repair, particularly in complex wounds like second-degree burns. These formulations maintain a moist environment, support hemostasis, and enable oxygen and nutrient exchange, thereby accelerating the healing cascade. At concentrations around 10% *w*/*w*, carrageenan gels have been shown to accelerate early-stage healing by supporting collagen matrix reorganization and glycosaminoglycan structuring, as confirmed by spectroscopic analyses [60].

When blended with other biomaterials like collagen or gelatin, carrageenan forms composite scaffolds that support cell proliferation, nutrient exchange, and extracellular matrix deposition, all essential for effective wound regeneration [61]. These scaffolds enhance fibroblast proliferation and extracellular matrix (ECM) deposition, while also supporting angiogenesis and re-epithelialization. For example, κ-carrageenan-PEO films reinforced with MgO nanoparticles demonstrated an enhanced thermal stability, tensile strength (up to 129 kPa), and swelling capacity (~52%), while maintaining low cytotoxicity and in vivo safety, positioning them as promising candidates for chronic wound care [62].

More advanced formulations, such as 3D nanocomposites incorporating TiO_2_ nanotubes, further enhance fibroblast activity and tissue integration, leading to faster and more complete wound closure in vivo. These characteristics position carrageenan-based constructs as valuable scaffolds in soft tissue engineering, offering both structural support and bioactive stimulation.

In the field of drug delivery, carrageenan’s functional groups enable the development of hydrogels, microspheres, films, and nanoparticles with controlled, sustained, and site-specific release capabilities.

The ability of κ-carrageenan to undergo ionic crosslinking in the presence of K^+^ or Ca^2+^ enables the formulation of ion-responsive hydrogels and gastroretentive systems with excellent mucoadhesive and floating properties [59]. In particular, κ-carrageenan-based hydrogels have been shown to extend the gastric residence time and maintain drug release over several hours, improving the therapeutic efficacy for locally or systemically acting drugs. Moreover, the incorporation of functional nanofillers, such as ferromagnetic particles, into carrageenan matrices has enabled the development of magnetically targeted drug delivery systems with enhanced biodegradability and responsiveness. Crosslinked carrageenan composites also show improved mechanical integrity and rapid disintegration in aqueous environments, making them suitable for both sustained and fast-dissolving formats, including hard capsules. Carrageenan’s mucoadhesive behavior is particularly advantageous in transmucosal delivery routes (e.g., oral, vaginal, buccal), where prolonged retention enhances drug absorption and localized action. As reported in the recent literature, mucoadhesion is influenced by the molecular weight, degree of sulfation, and formation of non-covalent interactions (electrostatic, hydrogen bonding) between the polysaccharide and mucins [61]. These mechanisms operate across different length and energy scales, from macroscale gel elasticity to nanoscale interfacial interactions, enabling the rational design of mucoadhesive devices.

Overall, κ-carrageenan represents a multifunctional platform material with demonstrated utility in both wound healing and drug delivery. Its ability to form thermoresponsive, ion-sensitive, and bioactive platforms, ranging from simple hydrogels to nanostructured composites, offers a highly customizable basis for developing next-generation biomedical systems tailored to specific clinical needs [63].

### 2.6. Ulvan

Ulvan, a sulfated polysaccharide extracted from green algae, is attracting growing interest as a marine-derived biopolymer due to its unique structural similarity to mammalian glycosaminoglycans (GAGs), such as heparan sulfate and chondroitin sulfate [64]. Its structure is mainly composed of 3-sulfated rhamnose linked to uronic acids (glucuronic and iduronic acid), xylose, and other minor sugars, forming repeating disaccharide units such as ulvanobiuronic acids A3s and B3s [64]. This structural homology underpins ulvan’s ability to mimic GAGs in modulating cell behavior, ECM interactions, and wound repair processes. Owing to its bioactivity, chemical versatility, and compatibility with cells, ulvan is being explored in multiple biomedical fields, particularly in controlled drug delivery, wound healing, and tissue engineering [65].

In drug delivery systems, ulvan has been incorporated into polymeric membranes and nanofiber systems with promising results. For instance, crosslinking with BDDE improves the mechanical strength and water resistance of ulvan-based membranes, while incorporation of drugs like dexamethasone enables both an initial burst and a sustained release, ideal for managing inflammation in wound settings. These scaffolds can maintain drug release over a period of up to 21 days, providing local control of inflammation and tissue repair stimuli. Similarly, ulvan–chitosan polyelectrolyte membranes support osteoblast adhesion and proliferation, leveraging their nanofibrous ECM-like architecture for effective drug incorporation and tissue targeting [66].

Although pure ulvan is difficult to electrospin due to limited solubility, it can be successfully processed into nanofibers when blended with synthetic polymers like poly(vinyl alcohol) (PVA), polycaprolactone (PCL), or polyethylene oxide (PEO). These hybrid nanofibers retain ulvan’s bioactivity and structural integrity: ulvan/PEO fibers, for instance, exhibit antithrombogenic properties useful in hemostatic wound applications, while ulvan/PCL fibers provide long-term scaffold support, ideal for tissue regeneration.

Moreover, ulvan has been employed in thermoresponsive nanogels, where its amphiphilic nature and functional sulfate groups facilitate encapsulation and controlled release of both hydrophilic and hydrophobic actives, including poorly soluble drugs such as curcumin, whose aqueous solubility was shown to increase by several orders of magnitude when loaded in acetylated ulvan nanogels [64].

In clinical settings, ulvan-based nanofibrous patches have shown efficacy in cutaneous wound healing. In a study involving 24 subjects undergoing cryosurgery for keloids, ulvan/PEO patches applied daily over 21 days demonstrated significant healing benefits. The nanofibers (47–621 nm, mean 282 ± 86 nm) led to significant improvements in healing parameters. Specifically, Patient and Observer Scar Assessment Scale (POSAS) scores decreased from 8 at day 7 to 2 by day 21, indicating near-complete restoration of the skin condition. Biophysical parameters such as hydration, transepidermal water loss (TEWL), erythema, and melanin content were significantly normalized in the treated group versus controls. Moreover, reductions in hemoglobin concentration, skin texture, and skin volume confirmed an anti-inflammatory effect and promoted tissue restoration.

Preclinical data further supports ulvan’s regenerative capacity. In SKH-hr2 murine burn models, topical application of ulvan gels at 5.0% *w*/*w* notably enhanced wound healing, especially during the later stages of recovery. The ulvan used in these studies had a molecular weight of ~1150 kDa and contained 47.5% sulfate and 40.4% carbohydrates, with high contents of bioactive rhamnose (25.9%) and uronic acids (17.9%). These structural features support its antioxidant, anti-inflammatory, and wound-healing activities, making it ideal for sustained-release wound dressing formulations [67].

The structural analogy with GAG is not only chemical but also functional. Ulvans derived from blade-like *Ulva* species, such as *U. australis* and *U. rigida*, exhibit a significantly higher iduronic acid content compared to those obtained from filamentous species like *U. compressa* or *U. prolifera* [65]. This characteristic leads them to more closely resemble endogenous GAGs such as heparin and dermatan sulfate, suggesting an even greater potential to mimic ECM–cell interactions. The iduronate content also influences the rheological behavior and the ability to form stable hydrogels, which are essential prerequisites for in situ biomedical applications, such as hydrogel scaffolds.

For all these reasons, ulvan is currently considered one of the most promising marine biopolymers for tissue engineering and regenerative medicine. Its ability to form hydrophilic three-dimensional structures, GAG-like structure, high water affinity, and biodegradability make it an ideal platform for controlled drug delivery and the development of bioactive scaffolds in both skin and bone tissue repair. However, variability in algal sources and extraction methods requires standardization to ensure efficacy and reproducibility.

In bone tissue engineering, ulvan has been successfully combined with PDLLA via CO_2_ sintering, yielding biocompatible scaffolds with excellent mechanical integrity. With functionalization to promote calcium phosphate deposition, these composites enhance osteogenic differentiation and mineralization, highlighting their potential as resorbable bone graft substitutes. Beyond regenerative medicine, ulvan also exhibits antioxidant, moisturizing, and emulsifying properties, rendering it valuable in cosmetic and nutraceutical formulations. Its rhamnosyl and glucuronic acid residues stimulate collagen synthesis and help counteract oxidative stress, while its amphiphilic character allows it to act as a natural stabilizer and emulsifier in the development of nanoparticles and complex delivery systems [68].

**Figure 7:**
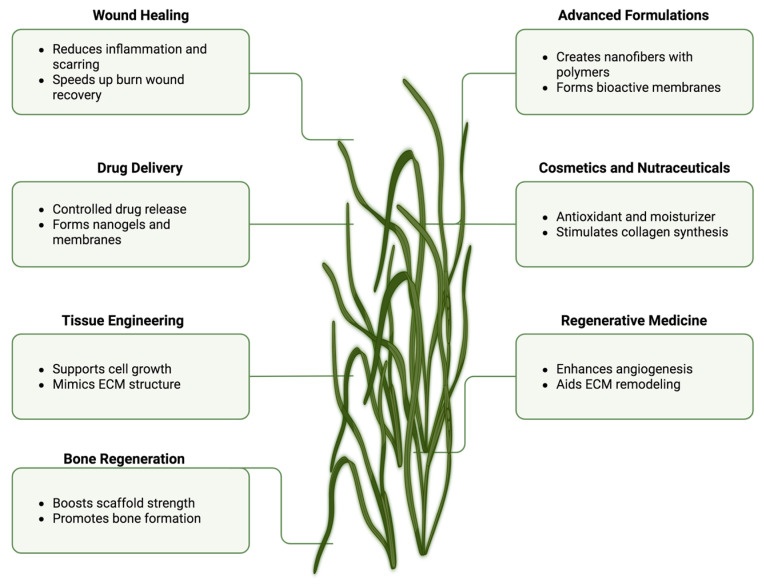
Schematic overview of ulvan’s key properties and biomedical applications (Created in BioRender. Chilwant, M. (2025) https://BioRender.com/5pj9a3a).

Altogether, ulvan’s multifunctional profile (Figure 7), encompassing tissue scaffolding, wound healing, drug delivery, and formulation enhancement, underscores its potential as a versatile marine biopolymer platform for next-generation biomedical technologies.

### 2.7. Hydroxyapatite

Hydroxyapatite (HA), a naturally occurring calcium phosphate mineral known for its structural role in bone, has proven highly effective in wound-healing biomaterials due to its bioactivity and chemical similarity to the mineral component of the ECM. When incorporated into marine biopolymer matrices such as carrageenan or alginate, HA forms composite materials that combine biocompatibility, bioactivity, and mechanical strength, supporting both the structural integrity of the wound site and the biological processes of regeneration.

Carrageenan, especially in its kappa form, forms hydrated and biocompatible gels, but lacks sufficient mechanical robustness to withstand physiological stresses. The incorporation of HA improves its compressive and tensile strength, enhancing its suitability for large or deep wounds and maintaining dimensional stability during tissue repair. Similarly, alginate, known for its exudate absorption and ionic crosslinking capacity, benefits from HA reinforcement, which increases rigidity while preserving flexibility, a key attribute for adapting to irregular wound geometries. These HA-enhanced composites also improve moisture retention and hemostasis, essential for acute wound care, while mimicking the ECM and providing mechanotransduction cues that regulate cell behavior and integration [69].

At the cellular level, HA functions as a temporary bioactive scaffold, offering a three-dimensional structure that facilitates fibroblast, keratinocyte, and endothelial cell adhesion, a critical first step in tissue regeneration. Its nanostructured surface promotes fibroblast proliferation and collagen matrix formation, while the gradual release of calcium and phosphate ions activates angiogenic pathways and stimulates the release of growth factors such as VEGF and TGF-β, further accelerating tissue repair. This process supports ECM remodeling, encouraging the deposition of new structural proteins like collagen and elastin, and is especially beneficial in chronic or non-healing wounds where ECM degradation compromises regenerative potential.

HA’s role extends beyond mechanical reinforcement: its porous and reactive nature enables the local delivery of antimicrobial agents, controlling infection and facilitating healing in diabetic ulcers, burns, and post-surgical wounds. Moreover, HA can serve as a sustained release reservoir for therapeutic compounds, enhancing localized bioactivity over time. In wounds involving connective tissue, its ability to promote dermal mineralization and stabilize the ECM contributes to long-term functional repair.

Overall, the integration of hydroxyapatite into marine biopolymer-based dressings creates structurally resilient, bioactive materials that support angiogenesis, tissue regeneration, infection control, and mechanical stability. These composites are particularly well-suited for the treatment of deep dermal injuries, chronic wounds, and post-operative defects, where both biological and mechanical restoration are essential for complete healing [70].

**Table 1:** Overview of fabrication processes for selected marine biopolymers used in biomedical applications. Each biopolymer undergoes a series of steps, including extraction, gelation, crosslinking, and final formulation, tailored to its physicochemical characteristics and intended use in drug delivery or wound healing.

Biopolymers	Fabrication Process	References
Chitosan	**Dissolution:** Chitosan is dissolved in an acidic solution, such as acetic acid, to disrupt its rigid crystalline structure and facilitate the formation of a viscous solution. **Nanoparticle Preparation:** Chitosan nanoparticles are synthesized using various techniques, including solvent evaporation, coacervation, or ionic gelation, where polyanions like sodium tripolyphosphate (TPP) facilitate nanoparticle formation through electrostatic interactions. **Crosslinking:** Chemical crosslinkers (e.g., glutaraldehyde, genipin) may be used to enhance stability and control the degradation rate. **Formulation:** The nanoparticles are then incorporated into various formulations such as hydrogels, films, or scaffolds, depending on the intended application (drug delivery, wound healing, etc.).	[71,72,73,74]
Alginate	**Gelation:** Alginate is typically dissolved in water, forming a gel-like structure. **Ionotropic Gelation:** Alginate forms gels when exposed to divalent cations (e.g., Ca^2+^). This process is used to create alginate beads, microcapsules, or hydrogels for drug delivery or tissue engineering. **Crosslinking:** Additional crosslinking agents (e.g., poly-L-lysine) can be introduced to modify the gel strength and stability. **Fabrication:** Cast into 3D scaffolds or employed in encapsulation systems.	[75,76,77]
Collagen	**Extraction:** Marine-derived type I collagen is extracted from various techniques but most prominently an acid extraction technique where acids (such as HCl and AcOH) hydrolyze the triple helix of collagen and solubilize its single chains in solution, where heavy-weight proteins are depolymerized into shorter peptides (0.3–8 kDa). **Gelation:** In aqueous solvents, collagen molecules can form collagen fibers. The self-assembly of collagen is an innovative approach to hydrogel formation. This process can be influenced by mechanical, physical, and environmental factors. **Crosslinking:** To increase the mechanical properties and stability of collagen matrices, chemical or physical crosslinking methods (e.g., glutaraldehyde or UV light) are applied. **Fabrication:** Cast into films, scaffolds, or hydrogels, which are then used in tissue engineering, wound healing, and cosmetic applications.	[47,48,49,78,79]
Fucoidan	**Extraction:** Fucoidan is extracted from brown seaweed through hot and pressurized aqueous and acidic extraction processes. Microwave-assisted extraction and ultrasound-assisted extraction are employed to prevent degradation of the cell wall and release the polysaccharide into the aqueous phase. **Purification:** The extracted fucoidan is purified by techniques such as dialysis, precipitation, or chromatography to remove impurities. **Gel Formation:** Fucoidan can be dissolved in water or combined with other gelling agents (e.g., alginate) to form hydrogels. **Cross Linking:** Fucoidan-based hydrogels may undergo physical or chemical crosslinking to control degradation rates and mechanical properties. **Fabrication:** Processed into nanoparticles, films, or scaffolds for use in drug delivery, wound healing, and biomedical applications.	[52,54]
Carrageenan	**Extraction:** From red edible seaweeds (e.g., alkaline treatment, cooking) **Precipitation**: Using alcohol or potassium chloride **Post-processing:** Washing, drying, and milling into powder	[80,81]
Ulvan	**Extraction**: From green algae (e.g., hot water, alkaline, enzyme-assisted, deep eutectic solvents) **Processing:** Into membranes/nanofibers via crosslinking (e.g., with BDDE) or blending with polymers (e.g., PVA, PEO, PCL) **Scaffold Formation:** Combination with PDLLA for scaffolds via CO_2_ sintering	[80,82,83]
Hydroxyapatite	**Synthesis:** From calcium and phosphate precursors (e.g., precipitation, hydrolysis, sol–gel) **Reaction Control:** Controlled mixing of solutions, adjusting pH/temperature **Thermal Treatment:** Calcination/sintering **Advanced Fabrication:** Hydrothermal, EPD, polymer-assisted for specific morphologies	[84,85]

**Table 2:** Summary of in vivo outcomes for marine-derived biomaterials used in wound healing and regenerative applications. The table distinguishes between preclinical and clinical studies, detailing the experimental model, formulation type, and key therapeutic results.

Biopolymer	Model	Formulation	Outcome	Study Type
Chitosan	Full-thickness rat model	Chitosan hydrogel with aloe vera	Complete wound closure in 14 days; enhanced epidermal thickness; reduced inflammation	Preclinical
Chitosan	Clinical (tooth extraction, patients with coagulation disorders)	Chitosan-based dressings	Reduced hemostasis time to 9.80 ± 15.49 min vs. 44.23 ± 22.41 min (control)	Clinical
Chitosan	Clinical	Chitosan sponge	Hemostasis time: 38 ± 8.7 s; faster clotting than controls	Clinical
Chitosan	In vivo rat model	Chitosan–graphene scaffold	87% reduction in clotting time vs. QuikClot	Preclinical
Alginate	Clinical (burn wounds)	Alginate dressings	Healing time: 7 ± 3.5 days vs. 14 ± 4.2 days (control)	Clinical
Alginate	Clinical (pressure ulcers)	Alginate dressings	74% of treated patients had ≥40% wound area reduction in 4 weeks vs. 42% in control	Clinical
Collagen	Sheep wound model	Collagen-based skin scaffolds (CBSSs)	Enhanced keratinocyte migration; increased granulation; reduced inflammation; upregulation of VEGF-A, hKER	Preclinical
Fucoidan	Murine full-thickness skin wounds	1.2% fucoidan topical gel	Faster wound closure from day 6; ~30% increase in granulation tissue; ~40% more collagen deposition	Preclinical
Fucoidan	In vivo angiogenesis model	Fucoidan	Enhanced CD31^+^/α-SMA^+^ vascular structures; increased angiogenesis markers (eNOS, VEGF, Nrf2, HIF-1α)	Preclinical
Ulvan	Clinical (24 subjects after cryosurgery)	Ulvan/PEO nanofiber patches	POSAS score from 8 to 2 over 21 days; normalized hydration, TEWL, erythema, and melanin	Clinical
Ulvan	SKH-hr2 murine burn model	5% ulvan gel	Improved healing at later stages; enhanced antioxidant and anti-inflammatory activity	Preclinical
Hydroxyapatite	Murine wound model	Gelatin-chitosan-CNC-HAp scaffold	50% faster healing; complete closure by day 7; delayed healing with crosslinked scaffold	Preclinical

## 3. Comparative Analysis of Marine Biopolymers

Marine-derived biopolymers are increasingly recognized as enabling materials in advanced biomedical applications thanks to their unique combination of biocompatibility, bioactivity, biodegradability, and functional versatility. Despite sharing a common natural origin, these biopolymers display diverse structural features that influence their solubility, processing, and therapeutic roles in contexts such as drug delivery and wound healing (Table 3).

Among them, chitosan, a cationic polysaccharide derived from the deacetylation of chitin, stands out for its mucoadhesive properties, pH-responsive drug release, and broad-spectrum bioactivity. It is soluble in mildly acidic solutions and undergoes enzymatic degradation by a lysozyme, making it highly suitable for biological systems. In drug delivery, chitosan enables the development of nanoparticles and hydrogels capable of controlled and targeted release, especially in mucosal applications. In wound healing, it promotes fibroblast proliferation, collagen deposition, and angiogenesis, while forming semi-permeable, moisture-retaining films that prevent microbial invasion. Chitosan’s regulatory status is well-established, with GRAS designation and FDA approval for wound and drug delivery system applications [73,86].

Similarly, alginate, a water-soluble anionic polysaccharide from brown algae, forms hydrogels through ionotropic gelation in the presence of divalent cations like calcium. It is biodegradable via enzymatic and ionic mechanisms and is highly biocompatible, though with limited inherent bioactivity. In drug delivery, alginate is appreciated for its ability to encapsulate hydrophilic molecules and modulate release kinetics, especially in oral and topical systems. In wound care, alginate excels due to its exceptional water retention, exudate absorption, and hemostatic activity. Its hydrogels create a moist environment favorable to healing and act as passive microbial barriers. As a gelling agent, alginate has a strong clinical track record, being widely used in FDA-cleared wound dressings [76,82].

Collagen, the main structural protein of the ECM, is insoluble in water but can be solubilized by acid or enzymatic treatment. It degrades naturally through collagenases, with excellent biocompatibility and low immunogenicity. As a scaffold material, collagen supports cell adhesion, proliferation, differentiation and angiogenesis [83]. These properties position collagen as a gold standard for tissue engineering and regenerative medicine applications, producing scaffolds that foster tissue growth and repair. In drug delivery, collagen matrices enable diffusion-controlled release of growth factors and peptides, while in wound healing, they accelerate granulation tissue formation and epidermal regeneration [87].

Gelatin, the thermally denatured form of collagen, is water-soluble and exhibits thermoresponsive sol–gel behavior near body temperature. It is biodegradable via proteases and retains many of the bioactive sites of native collagen. Gelatin’s excellent processability, film-forming capability, and compatibility with crosslinking agents make it ideal for the fabrication of microspheres, hydrogels, and 3D-bioprinted structures. In drug delivery, gelatin support fine-tuned release via enzyme-sensitive or thermally triggered mechanisms. In wound management, it facilitates moisture balance, gas exchange, and epithelialization, providing a flexible matrix for healing [77,87].

In contrast, fucoidan, a water-soluble, sulfated polysaccharide from brown seaweed, shows slow enzymatic biodegradation and a broad spectrum of bioactivities including anti-inflammatory, antioxidant, anticoagulant, and antiviral effects. Its amphiphilic nature and affinity for surface receptors (e.g., P-selectin) enable targeted drug delivery, especially in inflammation or cancer contexts. It is suitable for electrostatic or covalent incorporation into nanocarriers for dermal, mucosal, or systemic delivery. In wound healing, fucoidan enhances fibroblast activity, promotes collagen synthesis, and modulates local immune responses. It is often blended with chitosan or alginate to improve hydrogel biofunctionality [80,81].

Another promising material is carrageenan, particularly kappa-carrageenan from red algae, which is highly water-soluble and forms thermally stable gels in the presence of potassium or calcium ions. Although biodegradation is relatively slow, it is considered biocompatible and non-toxic. Its mucoadhesive, anti-inflammatory, and antiviral properties support its use in localized drug delivery platforms, including oral, vaginal, and topical formulations. In wound healing, carrageenan-based hydrogels promote fibroblast proliferation, collagen formation, and angiogenesis, particularly when reinforced with other materials like TiO_2_ or gelatin to improve mechanical strength [63].

Ulvan, a green algae-derived sulfated polysaccharide, is moderately soluble in water and often requires blending with polymers such as PVA or PCL for effective processing. It is biodegradable, mimics GAGs, and offers antioxidant, antithrombotic, and moisturizing properties. Even though its solubility and electrospinnability are limited, they improve when blended with compatible polymers. Ulvan has demonstrated efficacy in sustained-release membranes and bone-regenerative scaffolds, where it promotes collagen production, angiogenesis, and tissue mineralization [66,68].

Although not a polysaccharide, hydroxyapatite (HA) is frequently used in combination with marine biopolymers to improve mechanical strength, osteoconductivity, and biological signaling. HA is insoluble and only partially resorbable, but its porous and nanostructured form allows for the sustained release of ions (Ca^2+^, PO_4_^3−^) that promote angiogenesis, fibroblast activation, and ECM mineralization. HA-based composites with alginate or carrageenan are particularly effective in treating deep wounds, burns, and post-surgical defects, acting as temporary scaffolds that support tissue integration and healing [69,70].

In summary, marine biopolymers offer a remarkably diverse and modular toolkit for the design of drug delivery and wound-healing systems (Table 4). While chitosan leads in mucoadhesion and immune-responsive applications, alginate stands out for hydrophilic drug encapsulation and moisture management. Collagen and gelatin remain key players in tissue scaffolding and smart release, respectively, whereas fucoidan contributes valuable multifunctional bioactivity, particularly in inflammation- or oxidative-stress-driven disorders. Carrageenan provides structural versatility in localized therapies, and ulvan bridges antioxidant protection with regenerative engineering. Finally, hydroxyapatite, as a reinforcing agent, enhances the mechanical and biological profile of composite systems.

Taken together, these materials enable the development of customized, bioactive, and sustainable biomedical platforms, with significant potential for clinical translation in precision wound care, targeted drug delivery, and next-generation regenerative medicine.

## 4. Challenges and Future Direction

### 4.1. Production Challenges

Upscaling the manufacture of marine-derived polymeric nanoparticles from the laboratory to the industrial scale presents considerable challenges. While laboratory-scale synthesis routines such as ionic gelation and emulsification–solvent evaporation are commonly employed, these methods need to be adapted for industrial use. Key issues include maintaining a consistent particle size distribution, achieving a high drug encapsulation efficiency, and ensuring batch-to-batch reproducibility, all critical parameters for clinical and regulatory success [84].

Moreover, the raw material supply poses economic and logistical challenges. Many marine-derived biopolymers are extracted from regionally specific or seasonally limited resources, making them costly and sometimes unsustainable at scale [85]. In addition, industrial production demands advanced process automation, real-time monitoring, and compliance with GMP standards. Emerging solutions include continuous-flow bioprocessing, automated quality control platforms, and process analytical technologies (PATs), which can significantly enhance scalability, reduce variability, and lower production costs. For instance, continuous-flow systems have been successfully applied to the scalable production of chitosan nanoparticles via ionic gelation. By maintaining a constant flow of chitosan and crosslinking agents through microreactors or tubular flow systems, this method allows for precise control over mixing, the residence time, and particle formation, resulting in a uniform size distribution and reproducibility at an industrial scale. Collaborative efforts between academia and the industry are essential to develop scalable, cost-effective, and regulatory-compliant production pipelines [88].

### 4.2. Emerging Technologies

More recently, technological advances have opened up new routes to enhancing the performance of marine-derived polymeric nanoparticles. Techniques such as 3D printing and nanofabrication allow for unprecedented precision in the design of nanoparticles with tailored properties. For example, 3D printing enables the construction of complex scaffolds that can be customized to match individual anatomical or functional requirements, making them particularly suitable for tissue engineering and biomedical drug delivery. These scaffolds can also be functionalized with nanoparticles to provide targeted and controlled drug release in hybrid delivery systems.

Nanofabrication techniques such as lithography and self-assembly enable the engineering of nanoparticles with specific surface features, enhancing their binding selectivity to cellular targets and increasing therapeutic efficacy while reducing off-target effects. In parallel, microfluidic platforms are emerging as powerful tools for the scalable and reproducible synthesis of nanoparticles with precise control over size, morphology, and composition.

In addition, the development of stimuli-responsive (“smart”) marine-based materials is gaining traction. These materials can respond to external stimuli, such as pH, temperature, enzymes, or redox gradients, releasing the loaded active ingredient in a controlled and site-specific manner. For example, pH-sensitive chitosan-based nanoparticles have shown promise in delivering drugs preferentially to inflamed or infected wound sites, where the local environment is more acidic [74]. Similarly, alginate hydrogels incorporating temperature- or enzyme-responsive crosslinkers are under investigation for intelligent wound dressings that adapt to the wound environment.

Furthermore, the integration of biosensors within marine-derived wound dressings is an emerging frontier aimed at real-time wound monitoring. Recent studies have demonstrated the feasibility of embedding conductive components or sensor arrays into biopolymer-based hydrogels (e.g., chitosan or alginate matrices) to detect parameters such as pH, temperature, or bacterial metabolites, offering dynamic feedback for personalized wound management [89].

Together, these emerging technologies are paving the way for next-generation marine-derived biomaterials that combine regenerative, responsive, and diagnostic functions, maximizing therapeutic outcomes while supporting precision medicine approaches.

## 5. Sustainability Strategies in Marine Biomaterials

The increasing demand for marine-derived biomaterials raises critical concerns about the sustainability of sourcing practices and their potential impact on ecosystems and communities. Several complementary strategies have emerged to address these challenges, aiming to minimize ecological footprints while fostering responsible innovation in marine biotechnology. These approaches encompass bioconversion of by-products, development of synthetic analogues, optimization of extraction processes, controlled aquaculture, and ethical bioprospecting (Table 5).

### 5.1. Bioconversion of Seafood By-Products

Bioconversion involves the recovery and transformation of seafood processing residues, such as skins, bones, shells, and viscera, into high-value biomaterials like collagen, chitosan, and bioactive peptides. Utilizing biological processes such as microbial fermentation or enzymatic hydrolysis, this strategy not only reduces waste disposal costs and landfill use but also supports a circular bioeconomy by valorizing streams that would otherwise be discarded. Environmental benefits include a reduced carbon footprint and lowered resource extraction pressure, while social impacts involve the creation of new industrial sectors and green employment opportunities tied to sustainable biomass utilization [1,90]. Technological advances, including Ultrasound-Assisted Extraction (UAE), Enzyme-Assisted Extraction (EAE), Supercritical Fluid Extraction (SFE), Microwave-Assisted Extraction (MAE), Natural Deep Eutectic Solvents (DESs), Pulsed Electric Fields (PEFs), High Hydrostatic Pressure (HHP), and Membrane Filtration [91], have further improved the efficiency of protein, enzyme, and polysaccharide recovery from marine waste, thus facilitating their application in biomedicine, cosmetics, and food.

### 5.2. Development of Synthetic Analogues

Synthetic or bioengineered analogues are designed to mimic the structural and functional properties of marine biopolymers such as collagen, chitosan, and alginate, without relying on marine extraction. Produced via advanced chemical and materials engineering techniques, these analogues reduce the pressure on fragile marine ecosystems and help mitigate overharvesting risks. Their consistent quality, controlled production, and scalability address issues related to seasonal availability and batch variability of natural biomaterials. Furthermore, synthetic alternatives enable the tailoring of physical, chemical, and biological properties to specific applications, supporting the development of eco-designed and high-performance biomaterials [92].

### 5.3. Optimization of Extraction Processes

Extraction techniques for marine biopolymers can significantly influence both the environmental impact and material quality. The optimization of these processes focuses on maximizing the yield and purity while minimizing the use of hazardous solvents and energy. Innovations in green chemistry, including enzymatic extraction, ultrasound-assisted processing, and statistical design tools (e.g., response surface methodology), have enabled the efficient, low-impact recovery of biomolecules. As noted by Cameselle et al. [93], these optimizations contribute not only to cost-effectiveness but also to reduced generation of toxic residues, so marine biopolymer production becomes more aligned with sustainable industrial practices.

### 5.4. Controlled Aquaculture

Controlled aquaculture practices allow for the sustainable cultivation of marine species such as seaweed and crustaceans in regulated environments like tanks, ponds, or offshore farms. These systems enable precise management of environmental parameters (e.g., water quality, oxygen levels) and reduce the ecological damage caused by traditional wild harvesting or poorly managed farming [32]. Additionally, controlled aquaculture facilitates traceability and biosecurity, minimizing disease outbreaks and pollution. Environmentally, these approaches significantly decrease habitat degradation and overexploitation of wild stocks. Globally, aquaculture now accounts for 96.5% of the 31.2 million tons (fresh weight) of seaweed produced annually, with only 3.5% derived from wild harvesting, marking a significant shift toward sustainable cultivation. Asia dominates this production, with China, Indonesia, and other Asian countries contributing 47.9%, 38.7%, and 12.8%, respectively [94]. Socially, aquaculture provides stable livelihoods and opportunities for rural coastal communities when managed ethically and sustainably [95].

### 5.5. Ethical Bioprospecting

Ethical bioprospecting ensures that marine genetic resources are accessed and utilized in ways that uphold the rights of local communities and Indigenous peoples, particularly in biodiversity-rich regions. Central to this ethical framework is the concept of prior informed consent (PIC) of the country in which the resource is located, a principle enshrined in the Nagoya Protocol under the Convention on Biological Diversity. As of 2024, the Protocol has been ratified by over 140 countries, establishing a legal basis for fair and equitable sharing of benefits arising from the use of genetic resources [96].

This includes mechanisms such as financial compensation, capacity building, technology transfer, and infrastructural development, which are aimed at counteracting exploitative practices commonly associated with biopiracy. Ethical bioprospecting thereby fosters inclusive and collaborative partnerships among researchers, industry, governments, and community stakeholders, enhancing transparency, building trust, and facilitating the co-creation of sustainable and context-sensitive solutions.

Recent analyses of marine bioprospecting highlight the growing strategic interest in marine genetic resources, particularly in the context of international competition and emerging bioeconomies. According to Rusyaev and Orlov [97], the phenomenon of marine bioprospecting is characterized by its transdisciplinary nature, involving not only biological and technological challenges but also complex legal and ethical implications tied to global marine governance.

As highlighted by Lee et al. [98], the integration of equity and ethical accountability within research methodologies and commercialization frameworks constitutes a critical pillar of sustainable marine biotechnology. Their analysis underscores that the incorporation of traditional ecological knowledge, the acknowledgment of local and Indigenous customary systems, and the implementation of equitable benefit-sharing schemes represent foundational elements in the development of a socially responsible and ecologically balanced blue bioeconomy.

## 6. Regulatory and Safety Considerations

The assessment of safety, efficacy, and biocompatibility of marine-derived biomaterials, employed both as drug delivery systems and as clinical wound-healing devices, is a critical prerequisite for their clinical translation. Regulatory agencies such as the European Medicines Agency (EMA) and the U.S. Food and Drug Administration (FDA) require a comprehensive preclinical and clinical evaluation, including in vitro, ex vivo, and in vivo studies, to assess toxicity, immunogenicity, pharmacokinetics, and long-term effects [99]. In the European Union, products containing marine nanoparticles may fall under the scope of Regulation (EU) 2017/745 [100] on medical devices, particularly when classified as combination products, or under the EMA’s scientific guidelines such as the “Reflection paper on the data requirements for intravenous liposomal products” (EMA/CHMP/806058/2009 Rev. 2), which provide requirements for the physicochemical characterization, bioequivalence, and safety testing of nanoparticle-based formulations. These documents emphasize the importance of a detailed evaluation of critical quality attributes such as particle size distribution, surface charge (ζ-potential), stability, and release kinetics, in order to ensure batch-to-batch consistency and reproducibility. Biocompatibility evaluation is governed internationally by ISO 10993-1:2018 [101], which outlines the general principles for biological evaluation of medical devices within a risk management framework. This standard provides testing strategies for various biological endpoints, including cytotoxicity, sensitization, irritation, and systemic toxicity (ISO, 2018 [101]). For example, a material is typically considered biocompatible if it maintains at least 70% cell viability in cytotoxicity assays compared to untreated controls (ISO 10993-5:2009 [102]). However, specific quantitative thresholds often depend on the device classification, duration of contact, and intended clinical use.

In the United States, the FDA regulates nanomaterials under 21 CFR Part 312 (IND applications) and Part 314 (NDAs), requiring extensive characterization of particle behavior in biological systems and justification of safety margins (FDA, 2014 [103]). The FDA’s Biocompatibility and Toxicology Program also conducts regulatory science research on medical devices, promoting the adoption of New Approach Methodologies (NAMs) to improve predictivity, reduce reliance on animal testing, and align with the 3Rs principles (replace, reduce, refine). These methodologies include in vitro and in silico testing platforms integrated into regulatory assessments. The ICH M3(R2) guideline defines the nonclinical safety studies needed to support clinical trials and marketing authorization, including repeated-dose toxicity, genotoxicity, and reproductive toxicity studies (ICH, 2009 [104]). These studies are critical for de-risking early-phase trials, particularly for biomaterials with complex biological behavior like marine-derived polymers.

Although marine biomaterials such as chitosan and alginate are generally recognized as safe, their biological performance is highly dependent on physicochemical attributes. Preclinical evidence has shown that chitosan nanoparticles may induce cytotoxic effects depending on the particle size, surface charge, and degree of deacetylation [105]. For alginate, batch-to-batch variability in purity, molecular composition, and gelation behavior, due to differences in source organisms and extraction processes, can influence therapeutic consistency, while the presence of residual contaminants like polyphenols or endotoxins may provoke immune responses [106,107].

Several marine-derived products have already received regulatory approval, particularly in wound care applications. Examples include chitosan-based dressings such as ChitoFlex^®^ PRO and HemCon^®^ Bandage, as well as alginate-based products like Kaltostat^®^, Algisite™ M, and SeaSorb^®^ Ag, which are FDA-cleared or CE marked for clinical use. Additionally, marine-sourced fucoidan and collagen have been approved as food supplements or wound dressings in specific formulations. A summary of selected products, including the regulatory status and biomaterial composition, is provided in Table 6.

One of the main challenges in the regulatory evaluation of marine-derived biomaterials lies in their intrinsic biological and physicochemical variability, which is strongly influenced by species origin, environmental factors, and extraction methods. This variability poses a substantial barrier to the development of harmonized and reproducible toxicological protocols [10]. Consequently, a case-by-case approach is often required, emphasizing the need for flexible regulatory pathways and early engagement with competent authorities.

Strategies to overcome these challenges include the implementation of Quality by Design (QbD) principles, which allow for the systematic integration of risk-based control strategies from early development stages [88]. Furthermore, the use of predictive modeling, simulation tools, and artificial intelligence (AI) can significantly reduce the experimental burden, enhance material optimization, and improve preclinical success rates [108]. Ultimately, transparent dissemination of robust safety and efficacy data through peer-reviewed channels is vital to foster regulatory acceptance and public trust [89,99].

## 7. Conclusions

Marine-derived polymeric nanoparticles represent a promising and increasingly refined strategy for drug delivery and wound healing. Derived from sustainable sources such as chitosan (from crustacean shells) and alginate (from brown algae), these biopolymers offer distinctive physicochemical and biological properties that make them particularly suitable for biomedical applications. Their inherent bioactivity, biocompatibility, and biodegradability contribute to enhanced tissue regeneration while minimizing the risk of adverse effects.

In addition to their favorable safety profile, these marine-based nanoparticles exhibit remarkable versatility. Their ability to encapsulate and release a broad spectrum of therapeutic agents, including antibiotics, anti-inflammatory compounds, and growth factors, enables the development of targeted, controlled, and multifunctional treatment strategies tailored to different clinical needs.

As advancements in nanotechnology and materials science continue to evolve, the potential of marine-derived nanoparticles is expected to expand significantly. Emerging fabrication techniques and functionalization strategies are allowing for improved stability, site-specific delivery, and sustained therapeutic action, paving the way for next-generation bioactive systems in regenerative medicine and drug delivery.

## Figures and Tables

**Table 3:** Key properties of marine-derived biopolymers in biomedical applications.

Biopolymer	Solubility	Biodegradability	Bioactivity	Notable Functionalities
Chitosan	Soluble in acidic solutions	Enzymatically degraded (lysozyme)	Antimicrobial, hemostatic, immunomodulatory	Mucoadhesion, pH-sensitive release, supports tissue regeneration
Alginate	Water soluble; forms gels with divalent cations	Ion exchange and enzymatic degradation	Anti-inflammatory (mild)	Moisture retention, ionic crosslinking, wound exudate absorption
Collagen	Soluble after acid or enzymatic treatment	Biodegradable by collagenase	Promotes cell adhesion and proliferation	Structural ECM mimicry, angiogenesis promotion
Gelatin	Water soluble (thermoresponsive)	Biodegradable by proteases	Supports tissue growth	Easy to process, film-forming, thermo-sensitive for drug release
Fucoidan	Water soluble	Slow enzymatic degradation	Antioxidant, anti-inflammatory, antiviral	Stimulates fibroblasts, enhances immune response, and promotes probiotic synergy
Carrageenan	Soluble in hot water; forms gels depending on ionic strength (K^+^, Ca^2+^ ions)	Biodegradable; degradation rate varies by type (κ, ι, λ)	Moderate; can promote cell adhesion and proliferation	Gelling agent; wound dressing; drug delivery matrix; antiviral, antitumor, and anti-inflammatory potential
Ulvan	Water soluble; solubility increases with temperature and ionic strength	Highly biodegradable by ulvan lyases or gut microbiota	Immunomodulatory, antioxidant, and antimicrobial activities	Green algae-derived; sulfate-rich; forms hydrogels; potential for skin regeneration, vaccine delivery, and antioxidant wound healing
Hydroxyapetite	Poorly soluble in water; slightly soluble in acidic environments	Biodegradable; resorbability depends on crystallinity	High; supports osteointegration, bone bonding, and osteoinduction	Bone graft substitute; dental implant coatings; drug carrier; supports mineralization and cell adhesion; mimics natural bone composition

**Table 4:** Biopolymer drug release mechanisms, moisture retention and barrier properties, and biocompatibility and regulatory status.

Biopolymer	Drug Release Mechanisms	Moisture Retention and Barrier Properties	Biocompatibility and Regulatory Status
Chitosan	Mucoadhesive and pH-responsive systems; ionic crosslinking with polyanions (e.g., TPP) enables sustained release of bioactives	Forms semi-permeable films with moisture-preserving and antimicrobial barrier effects	High biocompatibility; GRAS status; FDA-cleared for wound dressings and DDS
Alginate	Ionotropic gelation with divalent cations enables controlled release kinetics, suitable for hydrophilic drug encapsulation	Crosslinked hydrogels retain exudate, promote a moist wound environment, and act as passive microbial barriers	Widely used in medical devices; strong clinical track record in wound management
Collagen	Carrier for growth factors and peptides; diffusion-controlled release within the fibrillar network	Supports granulation tissue, ECM deposition, and hydration; acts as a biological scaffold	Gold standard for biocompatibility; minimal immunogenicity; CE/FDA-approved
Gelatin	Protease-responsive matrix for controlled drug delivery; customizable via physical or chemical crosslinking	Maintains a moist microenvironment and enables gas exchange; semi-occlusive properties	High tolerability; extensively used in biofabrication and pharmaceutical formulations
Fucoidan	Controlled release via electrostatic or covalent incorporation in carriers; suited for anti-inflammatory, antioxidant, and anticancer agents	Enhances hydration; exhibits antioxidative and antimicrobial barrier effects	Demonstrates low cytotoxicity; immunomodulatory; promising for mucosal and dermal applications
Carrageenan	Ion-sensitive gelation enables controlled release; release rate tunable by ionic environment and gel strength	High water retention; forms semi-permeable gel barriers ideal for wound care and mucosal delivery	Generally high; widely approved in food/pharma (e.g., FDA GRAS), but usage in injectables under scrutiny
Ulvan	Forms hydrogels; allows diffusion-based or degradation-controlled drug release depending on formulation	Excellent moisture retention due to sulfate groups; forms protective films	High; low cytotoxicity; immunomodulatory properties; emerging biomaterial; not yet broadly approved but considered promising; requires case-specific validation
Hydroxyapetite	Surface adsorption/desorption and ion exchange; enables sustained release of charged drugs and biomolecules	Limited moisture retention; primarily acts as porous scaffold rather than barrier	Very high; mimics natural bone mineral; approved by FDA/EMA for bone grafts, dental implants, and some drug delivery devices

**Table 5:** Overview of strategic approaches for the sustainable exploitation of marine-derived resources, highlighting technological solutions and associated environmental and social benefits.

Strategy	Description	Environmental/Social Benefits
Bioconversion of seafood by-products	Recovery of by-products (e.g., skins, bones, shells) to obtain high-value biomaterials such as collagen, chitosan, and bioactive peptides.	Waste reduction, valorization of residues, and support for the circular economy.
Development of synthetic analogues	Design of synthetic biopolymeric materials that mimic the properties of marine biopolymers, reducing reliance on natural sources.	Reduced overexploitation, independence from resource seasonality.
Optimization of extraction processes	Improvement of extraction processes to minimize waste and increase yield, reducing environmental impact.	Greater sustainability of production processes, reduced toxic residues.
Controlled aquaculture	Sustainable cultivation of seaweed and crustaceans in controlled environments to reduce pressure on wild populations.	Low ecological impact, traceability, and environmental condition control.
Ethical bioprospecting	Ensuring fair compensation and benefit sharing with local communities that depend on marine resources.	Sustainable local development, increased acceptance and cooperation in research projects.

**Table 6:** Examples of marine-derived biomaterials that have obtained regulatory clearance for clinical use in wound care or drug delivery. The table reports the type of biomaterial, clinical application, and regulatory status, including FDA 510(k) clearances and CE markings under either the legacy MDD or current EU MDR frameworks.

Product Name	Marine Biomaterial	Clinical Application	Regulatory Status
ChitoFlex^®^ PRO	Chitosan	Hemostatic wound dressing (trauma, surgery)	FDA 510(k)
HemCon^®^ Bandage	Chitosan	Emergency hemostatic dressing	FDA 510(k)
Celox™ Gauze	Chitosan	Hemostatic agent (civilian and military use)	CE marked (EU MDR, Class III)
Kaltostat^®^	Calcium alginate	Absorptive wound dressing	FDA 510(k) + CE marked (MDD legacy)
Algisite™ M	Calcium alginate	Primary dressing for moderate-to-heavy exudate	FDA 510(k) + CE marked (MDD legacy)
SeaSorb^®^ Ag	Alginate + Silver	Antimicrobial dressing for infected wounds	CE marked (MDD legacy)
Maritech^®^ Fucoidan	Fucoidan (from brown algae)	Functional food ingredient, under wound care R&D	FDA GRAS (GRN No. 000626); EU Novel Food approved
MedSkin Solutions Collagen/Elastin Matrix	Marine collagen and elastin (from fish skin)	Dermal regeneration, chronic wounds	CE marked (EU MDR, Class III)
Collagen Matrix^®^ Wound Dressing	Fish-derived collagen	Temporary wound covering, absorbable scaffold	FDA 510(k)

## Data Availability

No new data were created or analyzed in this study. Data sharing is not applicable to this article.
